# Detection of the BRAF V600E Mutation in Colorectal Cancer by NIR Spectroscopy in Conjunction with Counter Propagation Artificial Neural Network

**DOI:** 10.3390/molecules24122238

**Published:** 2019-06-15

**Authors:** Xue Zhang, Yang Yang, Yalan Wang, Qi Fan

**Affiliations:** 1 School of Pharmacy, Chongqing Medical University, Chongqing 400016, China; zhangxue2017@139.com (X.Z.); yyang139@139.com (Y.Y.); 2 Department of Pathology, Molecular Medicine and Cancer Research Center, Chongqing Medical University, Chongqing 400016, China; wangyalan074@126.com

**Keywords:** near-infrared spectroscopy, counter propagation artificial neural network, detection, auxiliary diagnosis, BRAF V600E mutation, colorectal cancer, tissue, paraffin-embedded, deparaffinized, stained

## Abstract

This paper proposes a sensitive, sample preparation-free, rapid, and low-cost method for the detection of the B-rapidly accelerated fibrosarcoma (BRAF) gene mutation involving a substitution of valine to glutamic acid at codon 600 (V600E) in colorectal cancer (CRC) by near-infrared (NIR) spectroscopy in conjunction with counter propagation artificial neural network (CP-ANN). The NIR spectral data from 104 paraffin-embedded CRC tissue samples consisting of an equal number of the BRAF V600E mutant and wild-type ones calibrated and validated the CP-ANN model. As a result, the CP-ANN model had the classification accuracy of calibration (CAC) 98.0%, cross-validation (CACV) 95.0% and validation (CAV) 94.4%. When used to detect the BRAF V600E mutation in CRC, the model showed a diagnostic sensitivity of 100.0%, a diagnostic specificity of 87.5%, and a diagnostic accuracy of 93.8%. Moreover, this method was proven to distinguish the BRAF V600E mutant from the wild type based on intrinsic differences by using a total of 312 CRC tissue samples paraffin-embedded, deparaffinized, and stained. The novel method can be used for the auxiliary diagnosis of the BRAF V600E mutation in CRC. This work can expand the application of NIR spectroscopy in the auxiliary diagnosis of gene mutation in human cancer.

## 1. Introduction

**Figure 1:**
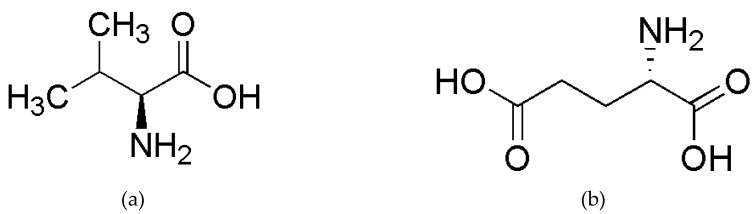
The structural formulas for valine (**a**) and glutamic acid (**b**).

Colorectal cancer (CRC) is one of the human malignant tumors with high incidence and mortality rates [1]. In particular, the mutations in CRC often make the treatment more difficult [2,3,4]. One of the most common mutations in CRC is the B-rapidly accelerated fibrosarcoma (BRAF) gene mutation, which involves a substitution of valine to glutamic acid at codon 600 (V600E) [5]. Figure 1 gives the structural formulas for valine and glutamic acid. The BRAF V600E mutation in CRC significantly reduces the efficacy of the drugs that are used in the treatment of patients with BRAF V600E wild type in CRC. The drug treatment regimen for patients with BRAF V600E mutant in CRC needs to be redesigned [6,7]. Therefore, it is crucial to detect the BRAF V600E mutation for the targeted therapy in CRC.

The typical methods for the clinical diagnosis of the BRAF V600E mutation in CRC are immunohistochemistry (IHC) in conjunction with microscopy [8], polymerase chain reaction (PCR) [9], and gene sequencing [10]. However, in IHC, the staining for target molecules that are associated with the BRAF V600E mutation is a multistep process. This process is frequently disturbed by many factors, resulting in staining failures, such as all negatives, all positives, too dark background, the positive control stained well but positive samples unstained or heterogeneous. Moreover, the diagnostic accuracy of microscopy is limited by the experience of pathologists. On the other hand, both PCR and gene sequencing are at least time-consuming and high-cost. Consequently, it is imperative to establish a sensitive, sample preparation-free, rapid, and low-cost method for the auxiliary diagnosis of the BRAF V600E mutation in CRC. 

Near-infrared (NIR) spectroscopy can be used to characterize the properties of an analyte containing the X-H groups (X = C, N, O, S). Typically, the vibration of one X-H group absorbs NIR light at several overtone frequencies, while the absorption intensity at a certain NIR frequency is the sum of the absorption intensities of a plurality of X-H groups. That is, the NIR absorption bands are seriously overlapping, so that NIR spectra are not directly interpreted and utilized. Thence, it is necessary to extract the information on the analytes from the NIR data for the sample by chemometric techniques [11,12,13]. NIR spectroscopy, assisted by chemometric techniques, is used to discriminate cancer from benign tumor, such as breast cancer [14], endometrial cancer [15], gastric cancer [16], and colorectal cancer [17], because it is easy-to-use, robust, inherently rapid (measuring a NIR spectrum in seconds), as well as nondestructive and low-cost [18,19].

Therefore, in this work, the feasibility of sensitive, sample preparation-free, rapid, and low-cost detection of the BRAF V600E mutation in CRC was explored with NIR spectroscopy and counter propagation artificial neural network (CP-ANN). The specific objectives are: (1) distinguishing the BRAF V600E mutant from the wild type by a CP-ANN model; (2) exploring the mechanism for NIR detection of the BRAF V600E mutation in CRC. This work can expand the application of NIR spectroscopy in the auxiliary diagnosis of gene mutation in human cancer. 

## 2. Results and Discussion

### 2.1. Samples

Table 1 lists 312 CRC tissue samples. Therein, the paraffin-embedded (Class 1) CRC sample is the most suitable for auxiliary diagnosis, because it is the most common form of pathological specimen storage. That is, the preparation of Class 1 samples is free. This means that the method of using Class 1 samples is top-priority, rapid, reagent-free, and nondestructive.

The deparaffinized (Class 2) and stained (Class 3) samples were used to explore the mechanism for NIR detection of the BRAF V600E mutation in CRC. However, the preparations of the Class 2 and Class 3 samples are both cumbersome and time-consuming. In addition, the samples of the combination of Class 2 with Class 1 samples (1:1) were named Class 2&1 samples. The Class 2&3 samples were named as similar to the Class 2&1 samples. Both Class 2&1 and Class 2&3 samples were also used to explore the mechanism for NIR detection of the BRAF V600E mutation in CRC.

The samples in each class consisted of an equal number of the BRAF V600E mutant and wild-type samples. The models calibrated while using an equal number of the BRAF V600E mutant and wild-type samples did not have classification biases that were caused by unequal numbers of samples in two subgroups. The number of validation samples was 30% of the number of calibration samples.

### 2.2. Spectral Acquisition

The NIR spectra of 312 CRC tissue samples were acquired while using the following means. The transflectance spectra, rather than transmission spectra, for the thin tissue samples were measured to increase the detection sensitivity. The sample signal intensity in the transflectance spectrum is twice that in the transmission spectrum, since the transflectance optical pathlength is twice the transmission one. Each sample was measured at three tissue locations, as the mutation may occur unevenly. The mutant and wild-type samples were alternately measured to avoid systematic errors that are caused by sequential measurement. Both 8 cm^−1^ resolution and 64 co-added scans were selected to obtain a spectrum with sufficient sample information and low noise in about 31.39 s. 

**Figure 2:**
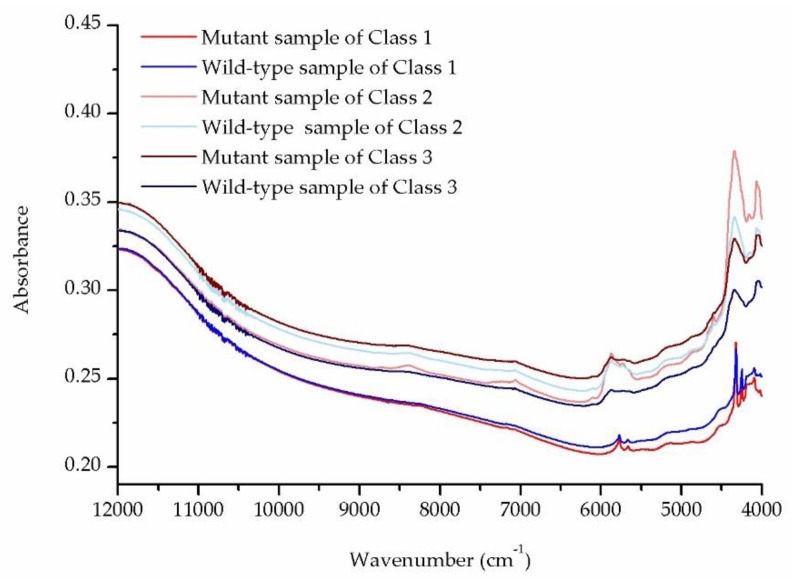
Mean near-infrared (NIR) transflectance spectra for colorectal cancer (CRC) tissue sections. Red, light red, and dark red represent, respectively, the mutant samples of Class 1, Class 2, and Class 3. Blue, light blue, and dark blue represent, respectively, the wild-type samples of Class 1, Class 2, and Class 3.

Figure 2 shows the mean NIR transflectance spectra for the mutant and wild-type samples of Class 1, Class 2, and Class 3. Red, light red, and dark red represent the mutant samples of Class 1, Class 2, and Class 3, respectively. Blue, light blue, and dark blue represent the wild-type samples of Class 1, Class 2, and Class 3, respectively. 

### 2.3. Data Processing

#### 2.3.1. Selection of the Spectral Preprocessing Strategy

Table 2 lists the vital preprocessing strategies, spectral subranges, numbers of PCs, numbers of neurons on each side, and corresponding model performances of the CP-ANN models built while using NIR data for the samples. The models, from Model 1 to Model 1.12, were built while using the same Class 1 samples, but changing preprocessing strategy, spectral subrange, number of PCs, and/or number of neurons on each side. Other models are similar to the above. As can be seen from Table 2, the models that were built using only mean centering (MC) have better model performances than those using other preprocessing strategies, respectively, for the models that were built using Class 1, Class 2, and Class 3 samples.

#### 2.3.2. Selection of the Spectral Subrange for Modeling

**Figure 3:**
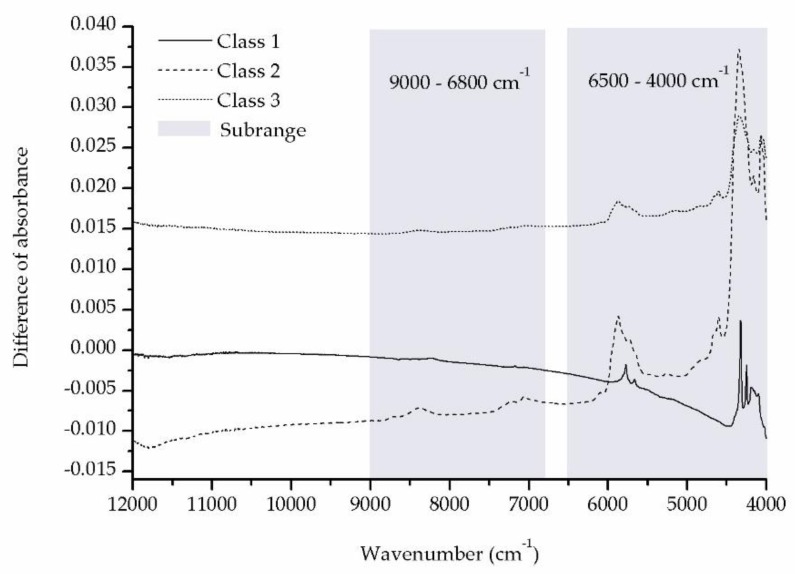
The differences between the mean spectra for the mutant and wild-type samples. The full, long dashed, and short dashed lines represent respectively Class 1, Class 2, and Class 3 samples.

Figure 3 indicates the differences between the mean spectra for the mutant and wild-type samples. The full, long dashed, and short dashed lines represent Class 1, Class 2, and Class 3 samples, respectively. On the full, long dashed, and short dashed lines, we can see significant changes in the two subranges 9000–6800 cm^−1^ and 6500–4000 cm^−1^.

The differences between the mutant and wild-type samples, in fact, are caused by the substitution of valine to glutamic acid. Figure 1 indicates that the largest structural difference between valine and glutamic acid is the difference between (CH_3_)_2_CH- in valine and -(CH_2_)_2_COOH in glutamic acid. Consequently, the spectral subranges 9000–6800 cm^−1^ and 6500–4000 cm^−1^ can be mainly attributed to the following overtones: the second overtones of CH_3_ and CH_2_ near 8696–8264 cm^−1^, CH near 8163 cm^−1^; the first overtones of CH_3_ near 5905 and 5872 cm^−1^, CH_2_ near 5680 cm^−1^, CH near 5882–5555 cm^−1^; the combination bands of CH_3_ near 7355, 7263, 4545–4500 and 4395 cm^−1^, CH_2_ near 7186 and 7080 cm^−1^, CH near 6944 cm^−1^; the combination bands of O-H in COOH near 4500–4000 cm^−1^ [20].

Table 2 shows the models that were built while using various spectral subranges. Model 1, Model 2, and Model 3 were built while using two spectral subranges 9000–6800 cm^−1^ and 6500–4000 cm^−1^. Model 1.10, Model 2.10, and Model 3.10 were built while only using one spectral subrange 9000–4000 cm^−1^. The two spectral subranges 9000–6800 cm^−1^ and 6500–4000 cm^−1^ were selected to build the detection model since Model 1, Model 2, and Model 3 had better model performances separately than Model 1.10, Model 2.10, and Model 3.10.

#### 2.3.3. Calibration and Validation of the CP-ANN Model

Principal component analysis (PCA) was used to reduce the redundant dimensionalities of the spectral data for the samples. The scores of the principal components (PCs, cumulative variance contribution rate exceeding 85.0%), as selected from both 9000–6800 cm^−1^ and 6500–4000 cm^−1^, were used as the inputs to the CP-ANN model. CP-ANN has the advantages of artificial neural network (ANN), such as nonlinearity, self-learning, self-organization, and self-adaptation [21]. Table 2 shows that the optimal structure of the CP-ANN model is 12 × 12, because the performances of the 12 × 12 model are better than the 10 × 10 one and nearly equal to the 15 × 15 one. 

**Figure 4:**
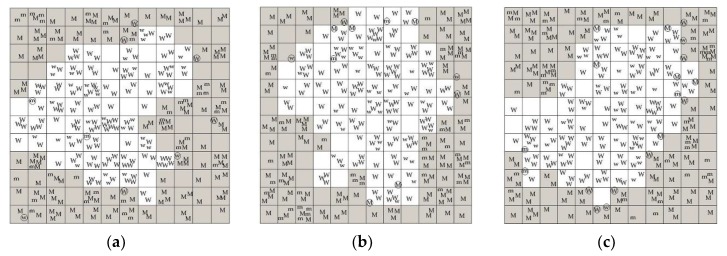
Projection maps for the 12 × 12 CP-ANN models: (**a**) Model 1; (**b**) Model 2; and (**c**) Model 3. The uppercase letter “M” and the lowercase letter “m” for the mutant samples, respectively, in calibration and validation; “W” and “w” for the wild-type samples, respectively, in calibration and validation; “○” for the samples assigned incorrectly; the gray region for mutant; the white region for wild type.

In Table 2, Model 1, Model 2, and Model 3 are optimal, respectively, for Class 1, Class 2, and Class 3 samples, because of the highest classification accuracies of calibration (CAC) and validation (CAV). Furthermore, Model 1, Model 2, and Model 3 have successively the best, medium, and worst classification accuracies. Figure 4a–c illustrate that the mutant and wild-type samples are assigned to the gray and white regions, respectively, by Model 1, Model 2, and Model 3, not only in the calibration (uppercase letter), but also in the validation (lowercase letter), although a few samples that are near the boundary are not correctly assigned. 

#### 2.3.4. Diagnostic Performances of the CP-ANN Model

Table 3 gives the diagnostic performances of five CP-ANN models that were sequentially built using an equal number of Class 1, Class 2, Class 3, Class 2&1, and Class 2&3 samples.

As can be seen from Table 3, each model shows a sensitivity of 100.0%. It can be inferred that the sample information in the acquired NIR transflectance spectra is sufficient for detecting the BRAF V600E mutation in CRC. That is, the structural differences between valine and glutamic acid on C-H, N-H, and O-H groups were characterized by NIR spectroscopy. In particular, a sensitivity of 100.0% is critical for auxiliary diagnosis, because it avoids missing the mutant.

In Table 3, Model 1, Model 2, and Model 3 have, respectively, medium, the best, and the worst specificities and accuracies. The probable cause is that the NIR spectra for the Class 1 samples are disturbed by the NIR absorption of paraffin; the NIR spectra for the Class 3 samples are disturbed by the NIR absorption of hematoxylin and eosin (HE). Moreover, the interference from paraffin is weaker than HE. However, the NIR spectra for the Class 2 samples are not disturbed by the NIR absorption of paraffin or HE. These inferences are supported by the following evidences. Model 2 is superior to Model 4 (built using Class 2&1 samples) and Model 5 (built using Class 2&3 samples) regarding the specificity and the accuracy; Model 4 and Model 5 are separately superior to Model 1 and Model 3. In addition, Model 4 is superior to Model 5.

On the other hand, HE is used to increase the color difference between the cancer and non-cancer tissues in pathological diagnosis. it is demonstrated that HE increases the absorbance difference between the mutant and wild-type samples since the color on the stained mutant tissue is darker than the color on the stained wild-type tissue, as shown in Figure 3. However, Model 3 (built using the HE-stained samples) has the worst diagnostic performances. A possible explanation is that HE interferes with the NIR detection and it does not increase the fundamental difference between the mutant and wild-type samples, that is, between valine and glutamic acid.

There are two kinds of differences in the calibration samples used in Model 4, as shown in Table 1. The first is the difference between the mutant and wild-type samples, i.e., the difference between valine and glutamic acid. The second is the difference between the deparaffinized and paraffin-embedded samples, i.e., the difference between no paraffin and paraffin. In fact, Model 4 distinguishes 80 calibration samples into two subgroups that are based on the difference between the mutant and wild-type, rather than between deparaffinized and paraffin-embedded. In other words, Model 4 detects the BRAF V600E mutation in CRC based on the difference between valine and glutamic acid in the deparaffinized and paraffin-embedded samples, rather than between no paraffin and paraffin in the mutant and wild-type samples. Similar results are obtained using the calibration samples in Model 5.

These findings suggest that the CP-ANN models built by the NIR data can detect the BRAF V600E mutation in CRC based directly on the fundamental difference between mutant and wild type, i.e., the difference between valine and glutamic acid, rather than among paraffin, HE, and nothing.

## 3. Materials and Methods 

### 3.1. Samples

312 CRC tissue sections of BRAF V600E mutant or wild type and their reference information were obtained from the Department of Clinical Pathology and the Molecular Medical Testing Center at Chongqing Medical University. The Ethics Committee of our university approved the collection and use of these specimens for current research. Informed consent was obtained from these patients.

These CRC tissue samples include three classes, as shown in Table 1. Class 1 is the paraffin-embedded sample on a glass slide, which is the most common form of pathological specimen storage; Class 2 is the deparaffinized sample between a glass slide and a coverslip; Class 3 is the HE-stained sample between a glass slide and a coverslip. Each class consisted of an equal number of the BRAF V600E mutant and wild-type samples.

The reference information on the BRAF V600E mutation in the CRC tissue sample was detected by real-time fluorescent quantitative PCR (RT-qPCR). The detection was performed on a Roche LightCycler 480 Ⅱ system (Roche, Basel, Switzerland) while using the Human BRAF Gene V600E Mutation Detection Kit (Wuhan YZY, China). The detection involved not only the PCR reaction, but also the PCR reaction for quality control (QC).

### 3.2. Instrument and Spectral Acquisition

The NIR spectra were measured while using a Nicolet iS50 FT-IR analyzer (Thermo Fisher Scientific, Waltham, MA, USA) that was equipped with an indium gallium arsenide detector and an integrating sphere. The instrument was controlled by OMNIC 9.2 software (Thermo Fisher Scientific, Waltham, MA, USA). 

A sample (glass slide up) was placed on the detection window of the integrating sphere and was covered by a lid with a gold inner top. The transflectance spectra for the samples were measured in the range of 12,000–4000 cm^−1^ while using the selected resolution and the selected number of co-added scans. The resolution was selected from 2, 4, 8, and 16 cm^−1^ to obtain sufficient sample information in a shorter time; the number of co-added scans was selected from 16, 32, 64, and 128 to reduce the noise in a shorter time. Each sample was measured at three tissue locations. The mutant and wild-type samples were alternatively measured. The background spectrum was measured, prior to the sample spectra, under the same conditions to eliminate any ambient interferences on the sample spectra. 

### 3.3. Data Processing

In the detection, the sample spectra were preprocessed by a preprocessing strategy that was selected from MC for subtracting the calculated mean of a variable from the spectral data, multiplicative scatter correction (MSC) or standard normal variate (SNV) for eliminating the interferences from granularity and compactness, derivative for deducting the background and separating overlapping signals, smoothing for denoising, and the combinations of various preprocessing techniques, as shown in Table 2.

The spectral subrange for modeling was selected from 12,000–4000 cm^−1^ based primarily on the differences in characteristic absorptions between the mutant and wild-type samples. 

Subsequently, the CP-ANN model was calibrated using the reference value of the calibration sample and the scores of the selected PCs in the spectral subrange of spectral data for the calibration sample. The three spectra per sample were all used in modeling. As shown in Table 1, Model 1, Model 2, Model 3, Model 4, and Model 5 were sequentially calibrated by 40 mutant samples and 40 wild-type samples of Class 1, Class 2, Class 3, Class 2&1, and Class 2&3. Next, Model 1, Model 2, and Model 3 were sequentially validated by 12 mutant samples and 12 wild-type samples of Class 1, Class 2, and Class 3. The structure of the CP-ANN model was selected from 10 × 10, 12 × 12, and 15 × 15 based on the model performances CAC, classification accuracy of cross-validation (CACV), and CAV.

The diagnostic performances of the CP-ANN model were evaluated with sensitivity, specificity, and accuracy. Sensitivity is defined as the ratio of TP/(TP + FN), where TP and FN are, respectively, the number of true positive (mutant) and false negative diagnostic results; specificity the ratio of TN/(TN + FP), TN and FP the number of true negative (wild type) and false positive diagnostic results; accuracy the ratio of (TP + TN)/(TP + FP + TN + FN). In the calculation of sensitivity, specificity, and accuracy, the final diagnostic result for the sample was calculated as a wild-type sample when the three prediction results for three spectra per sample were all wild type; otherwise, as a mutant sample. In other words, the final diagnostic result for the sample was calculated as a mutant sample when at least one of three prediction results for three spectra per sample was mutant.

TQ Analyst 8.0 software (Thermo Fisher Scientific, Waltham, MA, USA) was used for spectral preprocessing, selection of the spectral subrange for modeling, and PCA. Matlab 8.0 software (The Math Works, Natick, MA, USA) was used for the calibration and validation of the CP-ANN model.

## 4. Conclusions

The NIR strategy on the basis of the principle different from the clinical diagnostic methods can be used for the auxiliary diagnosis of the BRAF V600E mutation in CRC. The NIR detection is directly based on the molecular differences between the BRAF V600E mutant and wild type, so that it is undisturbed by the factors affecting sample staining in IHC. When compared to the time-consuming and high-cost PCR and gene sequencing, the NIR detection is sensitive, sample preparation-free, inherently rapid, and low-cost. This research expanded the application of NIR spectroscopy in the auxiliary diagnosis of gene mutation in human cancer. In addition, when combined with our previous work, i.e., the NIR spectroscopy for the auxiliary diagnosis of CRC while using the paraffin-embedded samples [22], it is expected to simultaneously diagnose CRC and the BRAF V600E mutation using the NIR spectra for colorectal tissue.

## Figures and Tables

**Table 1:** The numbers of models calibrated and validated using 312 colorectal cancer (CRC) tissue samples.

Model Number	Class of Samples	Number of Calibration Samples		Number of Validation Samples	
		Mutant	Wild-type	Mutant	Wild-type
1	Class 1	40	40	12	12
2	Class 2	40	40	12	12
3	Class 3	40	40	12	12
4	Class 2&1	20&20	20&20	NA	NA
5	Class 2&3	20&20	20&20	NA	NA

Note: NA for not available.

**Table 2:** Vital preprocessing strategies, spectral subranges, numbers of principal components (PCs), numbers of neurons on each side, and corresponding model performances of the counter propagation artificial neural network (CP-ANN) models built respectively using NIR data for Class 1, Class 2, Class 3, Class 2&1, and Class 2&3 samples.

Model Number	Preprocessing	Spectral Subrange (cm^−1^)	Number of PCs/ Cumulative Variance Contribution Rate (%)	Number of Neurons on Each Side	Model Performances		
					CAC (%)	CACV (%)	CAV (%)
**1**	**MC**	**9000–6800, 6500–4000**	**6/100.0**	**12**	**98.0**	**95.0**	**94.4**
1.1	MSC + MC	9000–6800, 6500–4000	6/99.9	12	97.0	93.0	90.3
1.2	SNV + MC	9000–6800, 6500–4000	6/99.9	12	97.0	94.0	81.9
1.3	FD + MC	9000–6800, 6500–4000	6/98.8	12	93.0	86.0	88.9
1.4	SD + MC	9000–6800, 6500–4000	6/95.7	12	89.0	71.0	73.6
1.5	SGS + MC	9000–6800, 6500–4000	6/100.0	12	98.0	94.0	90.3
1.6	SGS + FD + MC	9000–6800, 6500–4000	6/99.1	12	94.0	88.0	90.3
1.7	NDS + FD + MC	9000–6800, 6500–4000	3/100.0	12	92.0	85.0	87.5
1.8	MSC + SD + MC	9000–6800, 6500–4000	6/ 96.0	12	90.0	74.0	77.8
1.9	SNV + NDS + FD + MC	9000–6800, 6500–4000	6/100.0	12	95.0	88.0	90.3
1.10	MC	9000–4000	6/100.0	12	98.0	94.0	91.7
1.11	MC	9000–6800, 6500–4000	6/100.0	10	97.0	94.0	88.9
1.12	MC	9000–6800, 6500–4000	6/100.0	15	98.0	96.0	88.9
**2**	**MC**	**9000–6800, 6500–4000**	**6/100.0**	**12**	**97.0**	**92.0**	**94.4**
2.1	MSC + MC	9000–6800, 6500–4000	6/100.0	12	94.0	85.0	79.2
2.2	SNV + MC	9000–6800, 6500–4000	6/ 99.9	12	89.0	83.0	83.3
2.3	FD + MC	9000–6800, 6500–4000	6/97.2	12	90.0	82.0	86.1
2.4	SD + MC	9000–6800, 6500–4000	20/84.6	12	NA	NA	NA
2.5	SGS + MC	9000–6800, 6500–4000	6/100.0	12	96.0	94.0	90.3
2.6	SGS + FD + MC	9000–6800, 6500–4000	6/97.8	12	92.0	88.0	81.9
2.7	NDS + FD + MC	9000–6800, 6500–4000	2/100.0	12	88.0	80.0	79.2
2.8	MSC + SD + MC	9000–6800, 6500–4000	20/80.6	12	NA	NA	NA
2.9	SNV + NDS + FD + MC	9000–6800, 6500–4000	3/100.0	12	90.0	85.0	87.5
2.10	MC	9000–4000	6/100.0	12	96.0	91.0	93.1
2.11	MC	9000–6800, 6500–4000	6/100.0	10	96.0	90.0	87.5
2.12	MC	9000–6800, 6500–4000	6/100.0	15	97.0	92.0	94.4
**3**	**MC**	**9000–6800, 6500–4000**	**5/100.0**	**12**	**95.0**	**88.0**	**93.1**
3.1	MSC + MC	9000–6800, 6500–4000	5/99.9	12	86.0	71.0	66.7
3.2	SNV + MC	9000–6800, 6500–4000	5/99.9	12	85.0	72.0	68.1
3.3	FD + MC	9000–6800, 6500–4000	13/85.5	12	90.0	77.0	79.2
3.4	SD + MC	9000–6800, 6500–4000	20/75.0	12	NA	NA	NA
3.5	SGS + MC	9000–6800, 6500–4000	5/100.0	12	93.0	89.0	90.3
3.6	SGS + FD + MC	9000–6800, 6500–4000	10/ 85.6	12	88.0	79.0	76.4
3.7	NDS + FD + MC	9000–6800, 6500–4000	2/100.0	12	90.0	82.0	77.8
3.8	MSC + SD + MC	9000–6800, 6500–4000	20/74.5	12	NA	NA	NA
3.9	SNV + NDS + FD + MC	9000–6800, 6500–4000	4/100.0	12	87.0	65.0	72.2
3.10	MC	9000–4000	5/100.0	12	95.0	88.0	87.5
3.11	MC	9000–6800, 6500–4000	5/100.0	10	93.0	89.0	86.1
3.12	MC	9000–6800, 6500–4000	5/100.0	15	95.0	89.0	88.9
**4**	**MC**	**9000–6800, 6500–4000**	**5/100.0**	**12**	**97.0**	**97.0**	**NA**
**5**	**MC**	**9000–6800, 6500–4000**	**6/100.0**	**12**	**95.0**	**90.0**	**NA**

Notes: MC for mean centering; MSC for multiplicative scatter correction; SNV for standard normal variate; FD for first derivative; SD for second derivative; SGS for Savitzky-Golay smoothing; NDS for Norris derivative smoothing; PC for principal component; CAC, CACV and CAV respectively for the classification accuracy of calibration, cross-validation and validation; NA for not available.

**Table 3:** The diagnostic performances of five CP-ANN models built sequentially using an equal number of Class 1, Class 2, Class 3, Class 2&1, and Class 2&3 samples.

Model Number	Diagnostic Performances		
	Sensitivity (%)	Specificity (%)	Accuracy (%)
1	**100.0**	87.5	93.8
2	**100.0**	95.0	97.5
3	**100.0**	82.5	91.3
4	**100.0**	92.5	96.3
5	**100.0**	85.0	92.5
